# Progressive Muscle Relaxation Therapy in Epilepsy Management: Its Influence on Stress, Fatigue, and Quality of Life

**DOI:** 10.1155/bn/1370445

**Published:** 2026-03-25

**Authors:** Serpil Özmen, Afife Yurttaş

**Affiliations:** ^1^ Narman Vocational High School, Atatürk University, Erzurum, Turkey, atauni.edu.tr; ^2^ Faculty of Nursing, Atatürk University, Erzurum, Turkey, atauni.edu.tr

**Keywords:** epilepsy, fatigue, progressive muscle relaxation exercises, quality of life, stress

## Abstract

**Abstract:**

The purpose of this study is to assess the impact of progressive muscle relaxation exercises on fatigue, stress, and quality of life among individuals with epilepsy. This study was conducted as a randomized controlled intervention, incorporating a control group and pretest and posttest assessments. Between July 2019 and April 2020, a total of 75 patients with epilepsy participated, with 35 assigned to the intervention group and 40 to the control group. Participants in the intervention group were instructed to perform progressive muscle relaxation exercises three times per week for 6 weeks. No interventions were provided to the control group. Data were gathered using the personal information form, the Perceived Stress Scale (PSS), the Fatigue Severity Scale (FSS), and the Quality of Life in Epilepsy Inventory (QOLIE‐31). The mean FSS total score of the patients in the intervention group decreased significantly compared to those in the control group after the intervention (*p* < 0.05). No statistically significant change was observed in the stress scores. Cognitive and social function scores of the QOLIE‐31 scale subdimensions of the patients in the control and intervention groups were found to be significant (*p* < 0.05). Cognitive and social function scores of the patients in the intervention group were higher than those in the control group. Progressive muscle relaxation exercises were determined that progressive relaxation exercises did not reduce stress in patients with epilepsy but had a positive effect on fatigue and cognitive and social function scores, which are subscales of quality of life. Progressive relaxation exercises may be proposed as a complementary nursing intervention for patients undergoing epilepsy treatment.

**Trial Registration:**

ClinicalTrials.gov identifier: 1370445

## 1. Introduction

Epilepsy is a chronic neurological condition defined by recurrent and heterogeneous seizures, exerting substantial physical, social, and psychological burdens on affected individuals [1]. It is estimated that there are 70 million people with epilepsy worldwide, with 90% of them living in developing countries [2]. Although the underlying mechanisms of epilepsy have not been fully elucidated, genetic factors, cerebrovascular accidents, central nervous system infections, traumatic brain injuries, neoplasms, and febrile conditions are recognized as potential contributors to its etiology [2]. The fundamental aim of epilepsy management is to enhance patients’ quality of life by minimizing seizure frequency. However, seizure occurrence and exacerbation are modulated not only by the pathophysiology of epilepsy but also by the pharmacological agents employed in its treatment and by stress‐related factors [3]. Frequent and recurrent seizures resulting from these factors not only lead individuals to perceive an irreversible loss of health and functional capacity in daily activities but also induce fatigue, thereby adversely influencing their quality of life [4, 5].

Currently, it is estimated that a significant proportion of patients with epilepsy frequently report experiencing subjective fatigue, which commonly results in decreased physical strength [6]. Research indicates that 10%–25% of patients with epilepsy experience fatigue, which has been attributed to anxiety, depression, and sleep disturbances associated with recurrent and frequent seizures [7–9]. The significant social and psychological effects of epilepsy cause patients to experience fatigue by creating a feeling of exhaustion in their daily lives, and this situation negatively affects their quality of life [9]. Research indicates that a range of nonpharmacological interventions, including yoga, behavioral therapy, music therapy, and muscle relaxation exercises, are employed in patients with epilepsy to alleviate fatigue and enhance quality of life [10–12].

The nursing discipline, which has assumed responsibility for the individual’s healthcare, requires holistic and humane care for the individual. Progressive muscle relaxation (PMR) Exercises, as a natural method that can be independently applied by nurses to relieve discomfort associated with anxiety, stress, fatigue, pain, and sleep‐related factors, constitute one of the effective therapeutic techniques that may be employed in care to enhance quality of life and comfort levels [13–15]. Additionally, it is listed under Code Number 6040 in the Nursing Interventions Classification (NIC) and is included in the list of interventions for problems such as anxiety, stress, depression, and fatigue [16]. PMR exercises constitute mind–body interventions that achieve whole‐body relaxation through the systematic and voluntary relaxation of major muscle groups [17]. PMR offers multiple benefits, including reducing the effects of stress and anxiety, enhancing physical and psychological well‐being, diverting attention from pain, alleviating fatigue, and promoting sleep [18–21]. The advantages of PMR exercises including their absence of adverse effects, rapid learnability, affordability, and ease of implementation without the need for specialized equipment contribute to their popularity among patients and support their widespread adoption as an independent nursing intervention in disease management [17–19]. Among individuals with epilepsy, PMR exercises are gaining prominence as a recommended form of behavioral therapy [22]. However, an extensive review of the literature demonstrated that there are no studies specifically assessing the effects of PMR exercises on stress, fatigue, and quality of life in individuals with epilepsy. For this reason, the present study was conducted to evaluate the effects of PMR exercises on stress, fatigue, and quality of life in patients with epilepsy.

## 2. Methods

### 2.1. Study Design

This research was designed as a randomized controlled intervention study incorporating a control group and employing pretest and posttest assessments.

### 2.2. Participants

**Figure 1:**
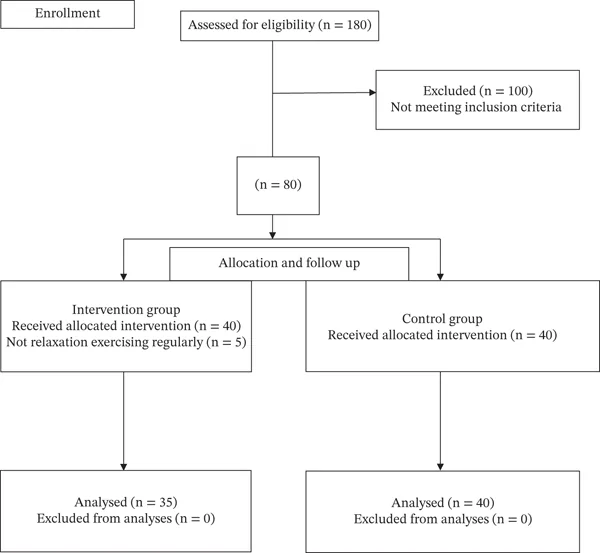
CONSORT study flow diagram.

The study population consisted of patients diagnosed with epilepsy who were referred to the neurology polyclinic and clinic of a university hospital in Erzurum, a city in the east of Turkey, between July 2019 and April 2020. The sample size for the study was calculated using the “G. Power‐3.1.9.7” program. Considering Cohen’s *d* = 0.77 moderate to large effect size previously reported by Pratiw et al., we estimated that a minimum sample size of 80 (40 in each group) would be required to detect an effect size of *d* = 0.77 for a two‐sample independent *t*‐test at 80% power and a 5% significance level. During the intervention, a total of five data were lost in the intervention group (*n* = 5) as predicted. The difference in sample size between the groups occurred due to voluntary participation and the accessibility of participants during the data collection process. Since some patients were excluded from the follow‐up due to the inclusion criteria, the study was completed with 75 epilepsy patients (Figure 1). The inclusion criteria were a confirmed diagnosis of epilepsy, age 18 years or older, absence of verbal communication barriers, literacy of the patient or a household member, absence of a diagnosed psychiatric disorder, physician confirmation that there were no physical limitations preventing exercise, ability to use technological devices, possession of a phone or computer with a voice recording function for exercise execution, and competency in using a phone or computer.

### 2.3. Randomization

Participants in the intervention and control groups were allocated according to the parity of the last digit of their barcode numbers, independent of age, marital status, or gender. Randomization was applied such that patients with even‐numbered barcodes were assigned to the intervention group, while those with odd‐numbered barcodes were assigned to the control group (Figure 1).

### 2.4. Data Collection Tools

#### 2.4.1. The Personal Information Form

The form has 16 questions. These questions include sociodemographic data and data about the disease such as gender, age, marital status, education level, income level, duration of epilepsy, other existing diseases, family history of epilepsy, seizure type, number of seizures, and seizure time.

#### 2.4.2. The Perceived Stress Scale (PSS)

The PSS was developed by Cohen et al. [23]. Consisting of 14 items in total, PSS is designed to measure how stressful a person’s life is perceived by certain situations. Participants evaluate each item on a 5‐point Likert‐type scale ranging from *Never* (0) to *Very often* (4). Seven of the items with positive expressions are scored in reverse. A high score indicates an excess of one’s perception of stress. The Turkish validity and reliability of PSS was performed by Eskin et al. [24]. The total Cronbach’s alpha value was 0.91 in the original scale [23]. In the present study, the total Cronbach’s alpha value was 0.83.

#### 2.4.3. The Fatigue Severity Scale (FSS)

The FSS, developed by Krupp et al. [25], has been validated for the Turkish population by Armutlu et al. [26]. The scale consists of nine items assessing fatigue, each rated on a 7‐point Likert scale. Total scores range from 9 to 63, with higher scores reflecting more severe fatigue. In the present study, the overall Cronbach’s alpha coefficient was 0.91.

#### 2.4.4. The Quality of Life in Epilepsy Inventory (QOLIE‐31)

A Turkish validity and reliability study of this scale was conducted by Mollaoğlu et al. in 2015 [27]. Quality of Life in Epilepsy Inventory (QOLIE‐31) includes a total of 31 items with 7 subscales, which are seizure worry (5 items), effects of drugs (3 items), energy/fatigue (4 items), emotional well‐being (5 items), cognitive function (6 items), social function (5 items), total quality of life (2 items), and an additional item evaluating total health condition. The scale is scored between 0 and 100, with a higher score showing a higher quality of life. The total Cronbach’s alpha value was 0.91 in the original scale [27]. In the present study, the total Cronbach’s alpha value was 0.90.

### 2.5. Data Collection Procedures

#### 2.5.1. Preapplication

Before starting the study, a pilot study was conducted with 10 patients who met the research criteria and agreed to participate in the study to evaluate the comprehensibility of the data collection forms. The patients included in the pilot study were not added to the study.

#### 2.5.2. The PMR Exercise Information Brochure

A PMR training brochure has been prepared for patients to perform muscle stretching–relaxation movements correctly. This brochure has photos of each of the muscle stretching movements and information about PMR.

Relaxation techniques were taught with the relaxation exercise CD.

#### 2.5.3. Relaxation Exercise CD

According to the Psychologists’ Association, this CD is composed of three parts. The first part, lasting 10 min, explains the objectives of relaxation. The second part, also 10 min, details the relaxation exercises using river sounds and verbal instructions. The final part, 30 min in duration, consists solely of relaxation music.

### 2.6. Intervention

#### 2.6.1. Interventions in the İntervention Group

Prior to data collection, the researchers informed the healthcare team at the hospital’s neurology outpatient clinic about the study. The pretest phase began with face‐to‐face interviews held in a private, quiet room in the outpatient clinic. During these interviews, the purpose of the study was explained, written consent was obtained, and a demographic information form was completed by the researcher using structured questions. Following this phase, all participants underwent baseline measurements (pretests) using the PSS‐14, FSS, and Quality of Life in Epilepsy Inventory(QOLIE‐31). Each data collection phase lasted approximately 45 min.

Following the pretest assessments, epilepsy patients who met the inclusion criteria were provided with detailed information about the intervention process. To ensure proper implementation of the technique, the researcher conducted an initial PMR training session with each patient individually. Patients in the intervention group were informed about the program’s duration, training content, and the materials to be used. A researcher‐prepared relaxation exercise information brochure and a relaxation exercise CD were provided. The researcher’s phone number was shared to provide support when needed.

For the PMR training sessions, patients were seated in a comfortable chair in a quiet environment. The relaxation exercises were first explained verbally by the researcher, then demonstrated using an audio recording, and finally practiced with the patient. Each session lasted approximately 45 min.

The PMR program was implemented 3 days a week under the researcher’s supervision. On the remaining days of the week, patients practiced the exercises independently at home. Home visits were made with the patient’s permission to reinforce skill acquisition, answer questions, and monitor compliance. Patients were contacted by phone the day before each supervised session to inform them of the session schedule.

The PMR intervention lasted 6 weeks and consisted of a total of 42 sessions. At the end of the 6‐week intervention, patients in the intervention group were readministered posttest measures of the PSS‐14, FSS, and QOLIE‐31 to assess the effects of the training.

The intervention was delivered using both the relaxation exercise information brochure and the relaxation exercise CD. The initial training sessions were conducted face‐to‐face by the researcher in the outpatient clinic to ensure accurate learning of the PMR technique. During the 6‐week intervention period, PMR sessions were carried out by the researcher 3 days per week during home visits, while the participants performed the exercises independently using the brochure and CD on the remaining days.

#### 2.6.2. Interventions in the Control Group

The first interview with the control group was conducted by the researcher in the neurology outpatient clinic. After informing the patients about the study, they completed a personal information form, the PSS‐14, the FSS, and the Quality of Life in Epilepsy Inventory (QOLIE‐31). Six weeks later, the PSS‐14, the FSS, and the Quality of Life in Epilepsy Inventory (QOLIE‐31) were administered again to the patients in the control group. Patients were given a relaxation exercise information brochure and a relaxation exercise CD. The patients were also informed about the importance of PGE use in their lives.

### 2.7. Data Analyses

The data obtained in the study were analyzed using the SPSS (Statical Package for the Social Sciences) 23.0 program. Percentages and mean values were used to analyze the sociodemographic characteristics of the patients. The *t*‐test was used to assess the normally distributed variables. For nonnormally distrubuted measurements, the Mannâ€“Whitney *U* test and the Wilcoxon test were used.

### 2.8. Ethical Considerations

The permissions were obtained from the ethics committee of a university’s faculty of nursing (2018‐10/3). The Turkish Psychological Association was used for PMR recordings in this study. Written permission was obtained from the Turkish Psychological Association to use the PMR CD. The participants were informed about the study’s procedures. Their written and verbal consent was obtained for the study. This study follows the Helsinki Declaration of Human Rights.

## 3. Results

Table 1 shows the comparison of the variable results of intervention and control group patients. Most of the patients in the intervention group were female (68.6%, *n* = 24), single (60%, *n* = 21), primary school (42.9%, *n* = 15), were diagnosed for 6–10 years (34.3%, *n* = 6–10 years), type of epilepsy was generalized (48.6%, *n* = 17), and was seizure frequency over the last Year 2 and above (65%, *n* = 26).

**Table 1:** Comparison of the variable results of intervention and control group patients.

Variables	Intervention group *n* = 35 (%)	Control group *n* = 40 (%)	Test value and significance
Gender			
Female	24 (68.6)	23 (57.5)	*χ* ^2^ = 0.9780.323
Male	11 (31.4)	17 (42.5)	
Age			
18–24	9 (25.7)	13 (32.5)	*χ* ^2^ = 0.9660.809
25–30	9 (25.7)	10 (25)	
31–35	5 (14.3)	7 (17.5)	
36 and above	12 (34.3)	10 (25)	
Marital status			
Married	14 (40)	21 (52.5)	*χ* ^2^ = 1.1770.555
Single	21 (60)	19 (47.5)	
Education level			
Primary school	15 (42.9)	19 (47.5)	*χ* ^2^ = 3.6370.303
Junior high school	5 (14.3)	11 (27.5)	
Senior high school	9 (25.7)	5 (12.5)	
University	6 (17.1)	5 (12.5)	
Duration of epilepsy			
1–5 years	8 (22.9)	14 (35)	*χ* ^2^ = 2.6090.456
6–10 years	12 (34.3)	9 (22.5)	
11–15 years	4 (11.4)	7 (17.5)	
15 and above	11 (31.4)	10 (25)	
Types of epilepsy			
Generalized	17 (48.6)	21 (52.5)	*χ* ^2^ = 4.2180.239
Simple partial	15 (42.9)	10 (25)	
Complex partial	1 (2.9)	2 (5)	
Myoclonic	2 (5.7)	7 (17.5)	
Seizure frequency over the last year			
None	4 (10)	6 (17.1)	*χ* ^2^ = 4.6880.096
1	10 (25)	15 (42.9)	
2 and above	26 (65)	14 (40)	

Just over half (57.5%, *n* = 23), the patients in the control group were female. More than half (52.5%, *n* = 21) were married, and education level was the most primary school in 47.5% of patients. The most common type of epilepsy was generalized (52.5%, *n* = 21), followed by simple partial (25%, *n* = 10). Seizure frequency over the last year of the patients in the control group was 2 and above (40%, *n* = 14) (Table 1).

Table 2 shows the comparison of the PSS‐14, FSS, and QOLIE‐31 total and subdimension pretest mean scores between the intervention and control groups. It was found that the difference between the mean scores of the scale total and subdimensions according to the groups was not statistically significant (*p* > 0.05) (Table 2).

**Table 2:** Comparison of PSS, FSS, and QOLIE‐31 total and subdimension pretest mean scores between groups.

Pretest	Group		*t*‐test	*p*
	Control	Experimental		
**PSS-14 total**	26.33 ± 7.53	26.17 ± 9.61	688.5	0.903 ^∗^
**FSS**	4.44 ± 1.76	4.66 ± 1.46	−0.595	0.554 ^∗^
**QOLIE-31 total**	49.97 ± 14.89	55.10 ± 10.86	564.0	0.149 ^∗∗^
Seizure worry	36.79 ± 28.65	46.52 ± 22.08	549.5	0.110 ^∗∗^
Total quality of life	57.19 ± 17.16	57.57 ± 17.45	698.5	0.987 ^∗∗^
Emotional well‐being	51.60 ± 19.57	50.06 ± 21.72	0.324	0.747 ^∗^
Energy/fatigue	44.13 ± 18.04	43.29 ± 15.72	0.679	0.823 ^∗∗^
Cognitive function	65.19 ± 16.73	67.06 ± 12.06	0.570	0.100 ^∗∗^
Effects of drugs	47.50 ± 26.89	56.74 ± 24.15	0.595	0.265 ^∗∗^
Social function	51.80 ± 18.43	51.49 ± 15.29	−1.843	0.069 ^∗^

*Note:* The bold text indicates scale names and subdimensions, not statistical significance.

∗*t*: independent sample *t*‐test.

∗∗*U*: Mann–Whitney *U*.

This shows the comparison of the posttest total and subdimension mean scores of the PSS‐14, FSS, and QOILE‐31 between the intervention and control groups. The difference in FSS scores between the patients in the intervention and control groups was significant.

Cognitive and social function scores of the QOLIE‐31 scale subdimensions of the patients in the control and intervention groups were found to be significant (*p* < 0.05). Cognitive and social function scores of the patients in the intervention group were higher than those in the control group. The differences between the other scale total and subdimension scores were not significant according to the groups (Table 3) (*p* > 0.05).

**Table 3:** Comparison of PSS, FSS, and QOLIE‐31 total and subdimension posttest mean scores between groups.

Posttest	Group		*t*‐test	*p*
	Control	Experimental		
**PSS-14 total**	27.03 ± 9.12	23.94 ± 7.51	560.5	0.136 ^∗^
**FSS**	4.71 ± 1.25	3.58 ± 1.26	3.909	**< 0.001** ^∗^
**QOLIE-31 total**	54.52 ± 14.76	60.45 ± 9.92	563.5	0.147 ^∗∗^
Seizure worry	45.91 ± 30.14	57.93 ± 18.71	571.5	0.172 ^∗∗^
Overall quality of life	57.19 ± 12.29	57.07 ± 15.63	680.5	0.835 ^∗∗^
Emotional well‐being	57.90 ± 22.81	57.83 ± 17.50	0.015	0.988 ^∗^
Energy/fatigue	48.38 ± 21.13	53.14 ± 14.51	560.5	0.137 ^∗∗^
Cognitive	60.97 ± 14.25	68.06 ± 15.46	459.5	**0.011** ^∗∗^
Medication effects	51.80 ± 28.00	60.63 ± 25.03	584.5	0.218 ^∗∗^
Social function	43.43 ± 21.5	59.91 ± 14.83	−2.080	**0.041** ^∗^

*Note:* The bold values represent statistically significant differences (*p* > 0.05).

∗*t*: independent sample *t*‐test.

∗∗*U*: Mann–Whitney *U* test.

## 4. Discussion

This investigation was undertaken to evaluate the impact of PMR techniques on stress, fatigue, and quality of life in patients diagnosed with epilepsy. The findings were analyzed in the context of extant literature. The study highlights that, in addition to pharmacological interventions, nonpharmacological strategies constitute a critical component in effective symptom management and seizure control among patients with epilepsy [28].

Although the etiology of fatigue in individuals with epilepsy is not fully known, it is stated that fatigue begins with the deterioration of the psychological and functional status of patients [7]. By using their independent functions, nurses play a role in coping with fatigue and this situation in epilepsy patients. Additionally, nursing interventions for fatigue in patients with epilepsy continue from the determination of fatigue to the end of treatment [8]. In the current study, a significant difference was found between the posttest FSS scores of the patients in the control and intervention groups (*p* < 0.05). In the study of Kütmeç Yılmaz and Kapucu, it was determined that PRE provided a significant decrease in the fatigue score averages of patients with COPD [29]. Dayapoğlu and Tan stated that PMR reduces fatigue in patients with multiple sclerosis [30]. Similarly, in the study conducted by Doğan et al., it was determined that PGE applied had a fatigue‐reducing effect [31]. Gisele et al. state that fatigue may be associated with uncontrollable seizures, deterioration of sleep quality, and depression in individuals with epilepsy. They also emphasized the importance of complementary therapies in addition to medical therapy to reduce fatigue in patients with epilepsy [32]. In the study of Çilliler and Güven, they stated that fatigue is a common symptom in patients with epilepsy and that complementary methods can be effective in reducing fatigue [33]. Michaelis et al. stated that relaxation exercises can reduce fatigue by increasing well‐being in patients with epilepsy [34]. Akgün Şahin and Dayapoğlu state that MR applied to patients with COPD reduces fatigue [35]. Moriya and Ikeda emphasize that PMR is important in coping with fatigue [36]. In this study, it was observed that the fatigue level decreased in the intervention group. This can be explained by the effects of regular PMR on the sympathetic nervous system and, on top of that, by providing relaxation of the skeletal muscles, making intense relaxation, reducing physical tension, and improving sleep quality.

The first condition of nursing care, which has an important place in providing stress management in individuals with epilepsy, is effective communication between the patient and the nurse. Effective communication creates a trusting relationship and enables the patient to participate actively in their own care [37]. In this study, no significant difference was observed between the posttest PSS‐14 scores of epilepsy patients in the control and experimental groups (*p* > 0.05). Polak et al. stated that PMR is a technique that improves seizure control. They state that the reason is related to the decrease in the stress levels of the patients [38]. Haut et al. reported that both practices reduced the number of seizures in their study, in which they used PMR and extremity exercises, while they stated that PGE was more effective in reducing stress [28]. Contrary to the above studies, in our study, it was determined that PMR administered to patients with epilepsy was not effective in reducing the stress level. As the reason for this, the often uncertain process of epilepsy can be shown. The uncertain process of epilepsy causes patients to experience many psychosocial problems. It can be thought that these problems may develop depending on the age of the patient; the duration of the epilepsy; the underlying etiology and neuropathology of the disease; the severity, frequency, and type of the seizures; the EEG discharge; the drugs and the inadequacy of the support experienced by the patient in his daily life; the chronic disease state; and the negative behaviors of the society towards [5].

Although the factors affecting the quality of life in individuals with epilepsy are not very clear, seizure control and severity are noted [39]. It is stated that factors such as stress, depression, fatigue, and sleep disorders that trigger seizures will affect the quality of life. It was observed that there was no significant difference (*p* > 0.05) between the posttest QOLIE‐31 scale total and seizure‐related anxiety, general quality of life, emotional well‐being, energy fatigue, and side effects of drug subdimension scores of the control and experimental group patients included in the study. Social function and cognitive function subdimension mean scores were observed to increase significantly in the experimental group compared to the control group (*p* < 0.05). As a result of this, it can be said that PMR did not affect the general quality of life between groups in our study, but only the scores of the social and cognitive function subdimensions. Michaelis et al. stated that therapies such as PMR and yoga could improve the quality of life by increasing well‐being in individuals with epilepsy [34].

McElroy states that alternative treatments such as PMR, yoga, and meditation have been used more frequently in epilepsy patients in recent years, and he states that these treatments can positively affect the quality of life [40]. Haut et al. think that relaxation exercises used as an adjunct treatment option can improve quality of life [41]. Ramaratnam et al., on the other hand, stated in their study that, in contrast to the above studies, relaxation exercises applied did not have a significant effect on the general quality of life of patients with epilepsy, except for the side effects of drugs and emotional well‐being, which are subdimensions of the QOLIE‐31 scale [42]. Talo and Bahçecioğlu Turan found that PMR exercises improved quality of life in epilepsy patients [43]. In our study, it was seen that PGE did not have an effect on the posttest intergroup QOLIE‐31 scale total score, but only on cognitive and social function, one of the subdimensions of the QOLIE‐31 scale. As the reason for this, it can be thought that the quality of life of individuals with epilepsy may be affected by physical and social functions, emotional, cognitive status, sleep and rest, energy, perception of health, seizures, other symptoms, and finally, physical and psychosocial factors related to being satisfied with their lives [27].

## 5. Limitations of the Study

This study has certain limitations. First, because it is based on the experiences of a single university hospital, the results may not be generalizable to the broader population of patients with epilepsy. Second, participation was limited to patients who were able to use technological devices. The unequal sample sizes between the intervention and control groups may limit the generalizability and comparability of the study findings.

## 6. Conclusion

PMR exercises were found to alleviate fatigue symptoms in individuals with epilepsy and to enhance the social and cognitive domains of the quality of life scale. Furthermore, findings from our study indicated that PMR was not effective in reducing stress levels among these patients. Based on these results, the following recommendations may be proposed:

− To regularly evaluate the severity of fatigue symptoms and quality of life in individuals with epilepsy.
− PMR exercises are an effective, easily applicable, and cost‐free nonpharmacological method into pharmacological treatment approaches.
− To provide PMR exercise training to patients with epilepsy through nursing staff and to establish an appropriate setting in neurology outpatient clinics and hospital wards for the practice of these exercise.
− To carry out studies with larger sample populations and extended follow‐up periods in order to more comprehensively examine the effects of PMR exercises on fatigue, stress, and quality of life.

## Author Contributions

Conception and design: S.Ö. and A.Y. Data collection: S.Ö. and A.Y. Analysis and result interpretation of data: S.Ö. and A.Y. Drafting of the manuscript: A.Y. Critical revision of the manuscript for important intellectual content: S.Ö. and A.Y. Statistical analysis: S.Ö. and A.Y.

## Funding

The study was funded by Atatürk Üniversitesi (10.13039/501100004951, TDK‐2019‐7144).

## Disclosure

All authors read and approved the final manuscript. This study was published as a full‐text conference paper in the 6th International Nursing and Innovation Congress, Istanbul, Turkey, October 22–23, 2022, pp. 522–523.

## Conflicts of Interest

The authors declare no conflicts of interest.

## Data Availability

The data that support the findings of this study are available on request from the corresponding author. The data are not publicly available due to privacy or ethical restrictions.

## References

[1] Akdağ G. , İlhan Algın D. , and Erdin O. , Epilepsy, Osmangazi Medical Journal. (2016) 38, 35–41, 10.20515/otd.88853.

[2] Mukhopadhyay H. K. , Kandar C. C. , Das S. K. , Ghosh L. , and Gupta B. K. , Epilepsy and Its Managament: A Review, Journal of Pharmaceutical Science and Technology. (2012) 1, 20–26.

[3] Erdoğan F. , Soyuer F. , Şenol V. , and Arman F. , The Impact of Fatigue on Quality of Life in Epilepsy Patients, Epilepsy Journal. (2006) 12, 21–26.

[4] Novakova B. , Harris P. R. , Ponnusamy A. , and Reuber M. , The Role of Stress as a Trigger for Epileptic Seizures: A Narrative Review of Evidence From Human and Animal Studies, Epilepsia. (2013) 54, no. 11, 1866–1876, 10.1111/epi.12377, 2-s2.0-84887237487. 24117321

[5] Görgülü H. and Fesci H. , Living With Epilepsy: The Psychosocial Effects of Epilepsy, Göztepe Tıp Dergisi. (2011) 26, 27–32.

[6] Chaudhuri A. and Behan P. , Fatigue in Neurological Disorders, Lancet. (2004) 363, no. 9413, 978–988, 15043967. 15043967 10.1016/S0140-6736(04)15794-2

[7] Kwon O. Y. and Park S. P. , Interictal Fatigue and Its Predictors in Epilepsy Patients: A Case-control Study, Seizure. (2016) 34, 48–53, 10.1016/j.seizure.2015.12.003, 2-s2.0-84958972461. 26723014

[8] Kwon O. , Ahn H. S. , and Kim A. J. , Fatigue in Epilepsy: A Systematic Review and Meta-analysis, Seizure. (2017) 45, 151–159, 10.1016/j.seizure.2016.11.006, 2-s2.0-85008235111. 28063374

[9] Ettinger A. B. , Weisbrot D. M. , Krupp L. B. , Coyle P. K. , Jandorf L. , and Devinsky O. , Fatigue and Depression in Epilepsy, Journal of Epilepsy. (2000) 11, 106–109, 10.1016/S0896-6974(97)00139-4, 2-s2.0-0031918565.

[10] Saxena V. S. and Nadkarni V. V. , Nonpharmacological Treatment of Epilepsy, Annals of Indian Academy of Neurology. (2011) 14, 148–152, 10.4103/0972-2327.85870, 2-s2.0-80054100220. 22028523 PMC3200033

[11] D’Alessandro P. , Giuglietti M. , Baglioni A. , Verdoni N. , Murgia N. , Piccirilli M. , and Elisei S. , Effects of music on seizure Frequency in institutionalized Subjects with severe/profound Intellectual disability and drug-resistant epilepsy, Psychiatria Danubina. (2017) 29, no. supplement 3, 399–404, 28953798. 28953798

[12] Puskarich C. , Whitman S. , Dell J. , Hughes J. , Rosen A. , and Hemann B. P. , Controlled Examination of Effects of Progressive Relaxation Training on Seizure Reduction, Epilepsia Journal. (1992) 33, 675–680, 10.1111/j.1528-1157.1992.tb02346.x, 2-s2.0-0026683030. 1628583

[13] Arslan Özkan H. and Bilgin Z. , Nursing Philosophical Essence Improvement and Healing Treatment Methods, Archives of Health Science and Research. (2016) 3, 191–200, 10.17681/hsp.49209.

[14] Charalambous A. , Giannakopoulou M. , Bozas E. , Marcou Y. , Kitsios P. , and Paikousis L. , Guided Imagery and Progressive Muscle Relaxation as a Cluster of Symptoms Management Intervention in Patients Receiving Chemotherapy: A Randomized Control Trial, PloS One. (2016) 11, no. 6, e0156911, 10.1371/journal.pone.0156911, 2-s2.0-84976551443. 27341675 PMC4920431

[15] Özdemir F. and Pasinlioğlu T. , The Effects of Training and Progressive Relaxation Exercises on Anxiety Level After Hysterectomy, New Journal of Medicine. (2009) 26, 102–107.

[16] Erdemir F. , Kav S. , and Yılmaz A. , Classification of Nursing Interventions (NIC), 2017, 6th edition, Nobel Medical Publishers.

[17] Kırca K. and Kutlutürkan S. , The Use of Progressive Relaxation Exercises in Cancer and the Treatment Process, Adıyaman University Journal of Health Sciences. (2020) 6, 258–267.

[18] Essa R. M. , Ismail N. , and Hassan N. I. , Effect of Progressive Muscle Relaxation Technique on Stress, Anxiety, and Depression After Hysterectomy, Journal Nursing Education Practice. (2017) 7, 77–86, 10.5430/jnep.v7n7p77.

[19] Kılıç N. and Parlar Kılıç S. , The Effect of Progressive Muscle Relaxation on Sleep Quality and Fatigue in Patients With Rheumatoid Arthritis: A Randomized Controlled Trial, International Journal of Nursing Practice. (2023) 29, no. 3, 13015, 10.1111/ijn.13015. 34569129

[20] Ramakrishnan M. and Chandran K. , The Effects of Progressive Muscular Relaxation Exercise Among Geriatric Patients With Psychiatric Illness, International Journal of Science and Research. (2015) 4, no. 4, 963–966.

[21] Krupinska K. and Kulmatycki L. , Effectiveness of Progressive Muscle Relaxation (Pmr) in Alleviating Psychophysical Disorders-A Systematic Review (1982-2012), GJRA-Global Journal for Research Analysis. (2014) 3, no. 10, 113–115.

[22] Rousseau A. , Hermann B. , and Whitman S. , Effects of Progressive Relaxation on Epilepsy: Analysis of a Series of Cases, Psychological Reports. (1985) 57, 1203–1212, 10.2466/pr0.1985.57.3f.1203. 3912787

[23] Cohen S. , Kamarck T. , and Mermelstein R. , A Global Measure of Perceived Stress, Journal of Health and Social Behavior. (1983) 24, no. 4, 385–396. 6668417

[24] Eskin M. , Harlak H. , Demirkıran F. , and Dereboy Ç. , Turkish Adaptation of the Perceived Stress Scale: Reliability and Validity Analysis, New Symposium Journal. (2013) 51, no. 3, 132–140.

[25] Krupp L. B. , Alvarez L. A. , LaRocca N. G. , and Scheinberg L. C. , Fatigue in Multiple Sclerosis, Archives of Neurology. (1988) 45, no. 4, 435–437, 3355400. 3355400 10.1001/archneur.1988.00520280085020

[26] Armutlu K. , Keser İ. , Korkmaz N. , Akbıyık D. İ. , Sümbüloğlu V. , Güney Z. , and Karabudak R. , Psychometric Study of Turkish Version of Fatigue Impact Scale in Multiple Sclerosis Patients, Journal of the Neurological Sciences. (2017) 255, 64–68, 10.1016/j.jns.2007.01.073, 2-s2.0-33947283199. 17337007

[27] Mollaoğlu M. , Durna Z. , and Bolayır E. , Validity and Reliability of the Quality of Life Scale (QOLIE-31) in Patients With Epilepsy in Turkey, Archives of Neuropsychiatry. (2015) 54, 289–295, 10.5152/npa.2015.8727, 2-s2.0-84941201798. PMC5353064 28360726

[28] Haut S. R. , Gursky J. M. , and Privitera M. , Behavioral Interventions in Epilepsy, Current Opinion in Neurology. (2019) 32, 227–236, 10.1097/WCO.0000000000000661, 2-s2.0-85071264228. 30694921

[29] Kütmeç Yılmaz C. and Kapucu S. , The Effect of Progressive Relaxation Exercises on Fatigue and Sleep Quality in Individuals With COPD, Holistic Nursing Practice. (2017) 31, no. 6, 369–377, 10.1097/HNP.0000000000000234, 2-s2.0-85031317128. 29028775

[30] Dayapoğlu N. and Tan M. , Evaluation of the Effect of Progressive Relaxation Exercises on Fatigue and Sleep Quality in Patients With Multiple Sclerosis, Journal of Alternatıve and Complementary Medicine. (2012) 18, no. 10, 983–987, 10.1089/acm.2011.0390, 2-s2.0-84867538376, 22967281. 22967281 PMC3469207

[31] Doğan S. , Tel H. , and Özkan M. , Effect of Relaxation Exercise on Fatigue, Depression and Level of Quality of Life in Diagnosed With Breast and Colorectal Cancer Within Patients Under Adjuvant Chemotherapy, Gevher Nesibe Journal of Medical & Health Sciences. (2022) 5, no. 8, 133–145, 10.46648/gnj.143.

[32] Gisele S. M. , Neves L. , and Gomes M. M. , Fatigue in Patients With Epilepsy and Its Association With Depression and Sleep Qualit, Arquivos de Neuro-Psiquiatria. (2013) 71, no. 8, 533–536, 10.1590/0004-282X20130075, 2-s2.0-84883438256. 23982011

[33] Çilliler A. E. and Güven B. , Inter-Ictal Fatigue and Antiepileptic Drugs in Patients With Epilepsy, Acta Neurologica Belgica. (2021) 121, no. 1, 107–111, 10.1007/s13760-020-01292-8, 32043217. 32043217

[34] Michaelis R. , Tang V. , Wagner J. L. , Modi A. C. , LaFrance W. C. , Goldstein L. H. , Lundgren T. , and Reuber M. , Psychological Treatments for People With Epilepsy, Cochrane Database of Systematic Reviews. (2017) 10, CD012081, 10.1002/14651858, 2-s2.0-85018168814. 29078005 PMC6485515

[35] Akgün Şahin Z. and Dayapoğlu N. , Effect of Progressive Relaxation Exercises on Fatigue and Sleep Quality in Patients With Chronic Obstructive Lung Disease (COPD), Complementary Therapies in Clinical Practice. (2015) 21, no. 4, 277–281, 10.1016/j.ctcp.2015.10.002, 2-s2.0-84947561063. 26573455

[36] Moriya R. and Ikeda N. , A Pilot Study of the Effects of Progressive Muscle Relaxation on Fatigue Specific to Multiple Sclerosis, British Journal of Neuroscience Nursing. (2013) 9, no. 1, 35–41, 10.12968/bjnn.2013.9.1.35.

[37] Buelow J. , Miller W. , and Fishman J. , Development of an Epilepsy Nursing Communication Tool: Improving the Quality of Interactions Between Nurses and Patients With Seizures, Journal of Neuroscience Nursing. (2018) 50, no. 2, 74–80, 10.1097/JNN.0000000000000353, 2-s2.0-85044050164. PMC5882248 29505437

[38] Polak E. L. , Privitera M. D. , Lipton R. B. , and Haut S. R. , Behavioral Intervention as an Add-On Therapy in Epilepsy: Designing a Clinical Trial, Epilepsy & Behavioral. (2012) 25, no. 4, 505–510, 10.1016/j.yebeh.2012.09.012, 2-s2.0-84871825588. 23153715

[39] Baybaş S. and Dirican A. C. , Bora İ. , Yeni N. , and Gürses C. , Quality of Life in Patients With Epilepsy, Epilepsy, 2008, 1st edition, Nobel Medical Publishing House, 727–733.

[40] McElroy C. , Alternative Approaches to Epilepsy Treatment, Current Neurology and Neuroscience Reports. (2009) 9, 313–318, 10.1007/s11910-009-0047-0, 2-s2.0-67651227572. 19515284

[41] Haut S. R. , Lipton R. B. , Cornes S. , Dwivedi A. K. , Wasson R. , Cotton S. , Strawn J. R. , and Privitera M. , Behavioral Interventions as a Treatment for Epilepsy: A Multicenter Randomized Controlled Trial, Neurology. (2018) 90, no. 11, e963–e970, 10.1212/WNL.0000000000005109, 2-s2.0-85046888097. 29444968

[42] Ramaratnam S. , Baker G. A. , and Goldstein L. H. , Psychological Treatments for Epilepsy, Cochrane Database of Systematic Reviews. (2008) 16, no. 3, 10.1002/14651858.CD002029.pub3, 2-s2.0-55049132565. 11687134

[43] Talo B. and Bahçecioğlu Turan G. , Effects of Progressive Muscle Relaxation Exercises on Patients With Epilepsy on Level of Depression, Quality of Sleep, and Quality of Life: A Randomized Controlled Trial, Seizure: European Journal of Epilepsy. (2023) 105, 29–36, 10.1016/j.seizure.2023.01.002, 36702017. 36702017

